# Women in the Profession

**Published:** 1877-10

**Authors:** A. J. Howe


### Miscellaneous.

### Women in the Profession.

A. J. Howe, M. D.

Upon this movement the *Medical Times and Gazette* says editorially: "we hold that, as women are bent on taking up medicine as a specially suitable and desirable vocation, notwithstanding all the experience, the advice, and warning to the contrary,—and as Parliament has in its wisdom decided that, so far as the laws are concerned, women shall have their wish in this respect,—it is neither wise, politic, nor expedient to place or leave in their path any unusual difficulties. We have now nothing more to say against their working out the experiment they are bent upon, except that we fear and believe they are making a pitiable mistake. We can not honestly congratulate the women on their victory, for we hold that it must lead to disappointment and disaster. Many women may very probably succeed in entering the ranks of the profession, and here and there one or two may obtain such success as will repay them, and justify their choice of the medical profession as a life career. But for the great majority we can but fear that only disappointment and failure await them, either before, or, still worse, after their mere student-days; and it is surely a melancholy thing to watch an experiment that we believe must in a very large proportion if not in a vast majority of instances, end in wasted means, wasted years, and blighted hopes. The women, however, will not be content without trying such an experiment, and it seems to be decreed that they shall have their wish."

While I am here to plead the rights of woman in the profession, I will take the opportunity to remark that proper restraints should be exercised by those who conduct the matriculation of women in our medical colleges. The age of applicants should be mature, and the literary qualifications respectable, or equal to the average of males who enter medical colleges. There is no place in the profession for the Dr. Mary Walkers, and such characters as seek notoriety at the expense of decency. As a general thing maidens and widows of moderate means will constitute a large proportion of those who desire to enter our medical colleges, the object of such being to better their pecuniary condition. It is unreasonable to suppose that the marriageable daughters of well-to-do families will sacrifice their chances in the matrimonial lottery for the less atttractive career of female physicians. If we are to admit women to the medical profession, we would prefer the very best of the sex, but not many such can we hope to obtain. Occasionally a woman brought up in wealth and refinement will, under a frenzied zeal to do good in the world, choose the study and practice of medicine as a vocation, and pursue it with the ardor peculiar to her nature; but such constitute an exception to the rule. And, unfortunately for the cause, quite a number have passed through our medical schools who do not possess a creditable education in English studies, and who were too weak minded to master the great truths and principles of medicine. They have barely squeezed through a loosely conducted examination, and entered upon a professional career where a vigorous rivalry starves the incompetent. They are looked upon with pity—a quality which does not furnish a doctor with patronage. A practitioner must possess qualities which inspire respect and admiration in order to be successful. A female physician may act as nurse, and command the attention of the best families. but unless she possess unusual tact and talents, she can not become their medical adviser. A physician never reaches the inner courts of the best people, unless he be equal to the best in mental and social qualities. A female physician of culture and lady-like manners, espeially if she has sprung from one of the "first families," may easily command a desirable patronage.

Female physicians are generally too impulsive in their nature, to patiently wait year after year for a lucrative practice. They desire to carry the world by storm, and pocket great fees before they have earned the right to the rewards of persevering labor. A young female graduate once remarked in my presence, that if she could not make a fortune out of medicine in five years, and take a trip to Europe, she would give up the profession in disgust. How often it is that a medical man of more than average ability does not obtain a lucrative practice until he is fifty years old. If a woman entered the profession at thirty, would she be willing to trudge along for twenty years without securing a paying practice ? I am afraid the less resolute and persevering of female practitioners would get discouraged before that time, and conclude they had mistaken their calling. I know that I shall be accused of presenting the dark side of the theme under discussion, yet those determined to enter the profession as female physicians will in time thank me for it.

It has been a question whether the average woman possesses the physical ability to practice medicine in town and country; but as the physique of males is not taken into consideration when they matriculate, some being smaller and weaker than the average woman, no sound argument on the subject can be sustained. Some women are powerful enough to drive a Concord coach over the rough roads of the Rocky Mountains. It is not expected that slim, slight, and slender girls of "sweet sixteen" are going to engage in the study and practice of medicine, but the physically and mentally strong. No woman who is below the average of her sex should receive any encouragement to enter the medical profession. Should any such attempt the career, they would surely and signally fail. If any man in the profession thinks that he is superior to the average woman—and there are plenty of such—it would be cowardly in him to fear the result of competition with female practitioners. If he be as much superior as he claims to be, he can afford to be generous towards her, even to the lending of a helping hand.

A school for nurses has just been opened in Paris. In this institution the instruction is imparted gratuitously, and generally in the evening when young women designing to become expert nurses can spend their time in learning without retrenching their incomes from daily labor. The teaching is conducted by young physicians and competent nurses; and diplomas are to be conferred upon such pupils as pursue these studies until fitted to nurse the sick. Of this movement the *Gazette Hebdomadaire* says: "This new institution responds to a real want. Let us have *instructed* nurses by all means, but instructed strictly in relation to the nature of the duties which ought to be demanded of them. God preserve us from *knowing* nurses, who have their opinions in medicine, even when only elementary, and their minds made up concerning hygienic measures! There are already too many of this sort: but intelligent and trained nurses are always needed to execute the commands of the physician."

In America women take up nursing as a means of earning a living, and never think that a course of instruction is needed to make them experts. If we had schools for the education of nurses, and statutory powers to grant degrees, I believe it would be better for those engaged in the calling, for a diploma would be a certificate which would pass as a guarantee of competency. In this country we have too many persons seeking skilled labor who have never served an apprenticeship; too many poor mechanics, and not enough good ones; too many shabbily educated doctors, and too few physicians of the highest order of talent; too many slatternly nurses, and a meager supply of those who have been through a system of training. Let some of those women who are desirous of benefiting the sick, and think there is no other way to accomplish the object except to become physicians, contemplate the feasibility of making themselves competent and reputable nurses.

In conclusion, I would respectfully ask those women who desire to enter the medical profession, if they realize the difficulties which beset their path; and if they would be content with a moderate and reasonable degree of success? I am glad that I have no sister who is ambitious to study medicine, but if I had, I would lend her a helping hand. If a woman intensely desires to become a physician, in the name of justice and equality let her have a fair chance to display her energies in acquiring a suitable education to enable her to efficiently execute her purpose. Perhaps she will not offer herself as a solicitor of professional patronage after she has secured medical honors. Many who have studied medicine do not practice what they have learned, but all such admit that what knowledge they have thus acquired is a source of pleasure and comfort to them.

The late Miss Harriet Martineau, in her Autobiography, which is now very eagerly read, expresses herself quite clearly upon the topic under discussion. She says: "Often as I am appealed to to speak or otherwise assist in the promotion of the cause of woman, my answer is always the same—that women, like men, can obtain whatever they show themselves fit for. Let them be educated,—let their powers be cultivated to the extent for which the means are already provided, and all is wanted or ought to be desired will follow of course. Whatever a woman proves herself able to do, society will be thankful to see her do,—just as if she were a man. If she is scientific, science will welcome her as it has welcomed every woman so qualified. I believe no scientific woman complains of wrongs."—*Eclectic Medical Journal.*

